# Salicylic acid: a key natural foundation for next‐generation plant defense stimulators

**DOI:** 10.1002/ps.70389

**Published:** 2025-11-24

**Authors:** Ruth Oussou, Sylvain La Camera, Cécile Marivingt‐Mounir, Jean‐François Chollet

**Affiliations:** ^1^ Institut de Chimie des Milieux et des Matériaux de Poitiers (IC2MP), Unité Mixte de Recherche CNRS 7285, Université de Poitiers Poitiers France; ^2^ Laboratoire Écologie & Biologie des Interactions (EBI) Unité Mixte de Recherche CNRS 7267, Université de Poitiers Poitiers Cedex 9 France

**Keywords:** sustainable agriculture, plant defense inducers, salicylic acid, bioprecursors, vectorized delivery systems

## Abstract

The field of crop protection is undergoing a major transition. The use of conventional biocidal compounds, which have dominated plant protection since the mid‐20th century, is now being questioned by societal demands for alternatives that are environmentally friendly and safe for human health. Consequently, new agronomic strategies are needed to ensure sustainable disease management. Among these, the activation of natural plant defenses against pathogens has emerged as a key approach and forms the central theme of this review. Salicylic acid (SA), a seemingly simple bifunctional aromatic compound, plays a pivotal role in plant immunity and in the establishment of systemic acquired resistance (SAR). However, its practical use is limited by rapid metabolism, compartmentalization, and potential phytotoxicity. To overcome these constraints, numerous SA analogs and derivatives have been developed to mimic or prolong its defensive action. A particularly promising, though still underexplored, approach involves the design of mobile bioprecursors and controlled‐release formulations capable of distributing throughout the plant and gradually releasing SA or related active species. Such approaches could sustain defense activation over extended periods while minimizing metabolic and environmental costs, paving the way for next‐generation plant defense stimulators. © 2025 The Author(s). *Pest Management Science* published by John Wiley & Sons Ltd on behalf of Society of Chemical Industry.

## Graphical abstract

Salicylic acid is emerging as a key natural molecule in sustainable crop protection, supporting diverse strategies to stimulate and sustain plant immunity for a greener agricultural future.

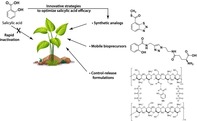

## 1 INTRODUCTION

Plants, as sessile organisms, are continuously exposed to a wide range of environmental stresses and interacting organisms, including pathogens such as viruses, fungi, and bacteria, which account for major crop losses worldwide.^1^ Pathogens can be broadly classified into necrotrophs, which destroy host cells and feed on their contents;^2^ biotrophs, which draw their nutrients from living cells;^3^ and hemibiotrophs, which are biotrophs for part of the infectious cycle and then become necrotrophs.^4^ To counter these threats, plants have evolved complex defense strategies, combining constitutive barriers with inducible responses that together form the plant immune system.^5^

This immune system comprises two major branches: PAMP‐Triggered Immunity (PTI) which provides basal defense, and Effector‐Triggered Immunity (ETI), which is typically stronger and more specific.^5^ Importantly, these local responses can lead to systemic acquired resistance (SAR), which primes distal tissues for enhanced protection. Such systemic signaling relies heavily on phytohormones, which integrate pathogen perception into long‐distance defense response. Among these, salicylic acid (SA) has emerged as a pivotal regulator of defense against biotrophic and hemibiotrophic pathogens.^6^ Recent studies further highlight SA's role as a central integrative hub, coordinating plant responses not only to pathogens but also to herbivores and other biotic challenges.^7^

The central role of SA in SAR has been extensively documented. Accumulation of SA leads to the induction of pathogenesis‐related proteins (PR), particularly PR1, and requires the key regulatory protein NPR1 (Non‐expressor of PR1), which undergoes redox‐dependent conformational changes that enable defense gene activation.^8^, ^9^ Genetic studies have further confirmed the importance of SA: plants expressing the bacterial *nahG* gene, which encodes a salicylate hydroxylase, are unable to accumulate SA and fail to establish SAR,^10^ whereas exogenous application of SA or its analogs restores defense capacity.^11^ Similarly, SA‐deficient mutant plants exhibit impaired resistance, underscoring the essential role of this hormone in plant immunity.^12^

Despite its central role, the direct use of SA as a crop protection tool faces important limitations. When applied exogenously, SA is often phytotoxic, rapidly metabolized, and compartmentalized, resulting in limited persistence and reduced long‐term efficacy.^13^ These drawbacks have stimulated the search for plant defense stimulators‐compounds, natural or synthetic, that prime or enhance plant resistance without directly targeting pathogens.^14^ Such compounds, including SA derivatives and structural analogs, represent promising alternatives to conventional agrochemicals. Used in a prophylactic approach, they can reduce disease incidence and contribute to sustainable crop protection strategies.^15^

This review first provides an overview of naturally occurring SA derivatives and their synthetic analogs that have been applied as defense inducers. We then highlight recent innovations designed to overcome the limitations of SA, including nanocomposites, conjugates, and novel delivery strategies that improve stability, mobility, and efficiency of SA‐based compounds. By emphasizing these next‐generation approaches, we aim to provide a forward‐looking perspective on how SA can serve as a natural foundation for developing more effective plant defense stimulators in agriculture.

## 2 THE PLANT INNATE IMMUNE SYSTEM: A SOPHISTICATED NETWORK AGAINST PATHOGENS

The plant immune system comprises two main branches: PAMP‐triggered immunity (PTI) and effector‐triggered immunity (ETI). PTI is based on the recognition of common elicitors, called microbe or pathogen‐associated molecular patterns (MAMPs or PAMPs), by transmembrane pattern‐recognition receptors (PRRs).^5^ Examples include bacterial flagellin or fungal chitin. This recognition activates basal defense signaling cascades that restrict pathogen invasion and provide broad, though unspecific, protection.^16^, ^17^

To circumvent PTI, pathogens have developed effectors that interfere with recognition and signaling, resulting in effector‐triggered susceptibility (ETS). Plants counteract this suppression through ETI which is mediated by intracellular proteins belonging to the NB‐LRR (Nucleotide binding‐leucine‐rich repeat) family associated with R genes. This specific recognition, described by Flor in the gene‐for‐gene theory,^18^, ^19^, ^20^ initiates strong defense response against the invading pathogen^17^ and is often associated with hypersensitive response (HR), a localized programmed cell death that restricts pathogen spread.

The co‐evolutionary dynamics between the plant's defense and pathogen effectors are illustrated by the ‘zigzag model’, which depicts successive cycles of PTI activation, effector‐mediated suppression, and renewed ETI recognition, leading to alternating phases of susceptibility and resistance.^5^

Defense responses induced by PTI and ETI include the rapid production of reactive oxygen species (ROS) by plant cells. These molecules damage pathogens, reinforce cell walls, and act as messengers for the activation of defense genes.^21^, ^22^ Such signals stimulate the production of antimicrobial proteins (PR proteins) and secondary plant metabolites such as phytoalexins, which further strengthen the defense arsenal.^23^, ^24^, ^25^ The HR, a hallmark of ETI, not only limits pathogen spread, but also contributes to the establishment of SAR.^5^, ^26^

SAR provides a broad‐spectrum and durable protection, often compared to immune memory in animals. Ross first demonstrated this phenomenon in 1961.^27^ Infection of the lower leaves of tobacco plants (*Nicotiana tabacum* L.) with tobacco mosaic virus (TMV) induced SAR, resulting in significantly reduced symptoms when the upper leaves were subsequently infected.^27^ The establishment of SAR is closely linked to SA accumulation in systemic tissues.^8^, ^11^, ^28^ However, grafting experiments indicate that SA itself may not be the mobile signal, but its accumulation in systemic tissues remains essential for the establishment of SAR.^29^, ^30^

## 3 SALICYLIC ACID: A MULTIFUNCTIONAL PLANT HORMONE

SA is one of the most studied phytohormones in plant defense, but its history goes back to its identification in willow (*Salix alba* L.) and its early medical use. SA was chemically synthesized in 1859 by Hermann Kolbe, and later acetylated to produce aspirin (acetylsalicylic acid) in 1897 by Felix Hoffmann.^8^

**Figure 1:**
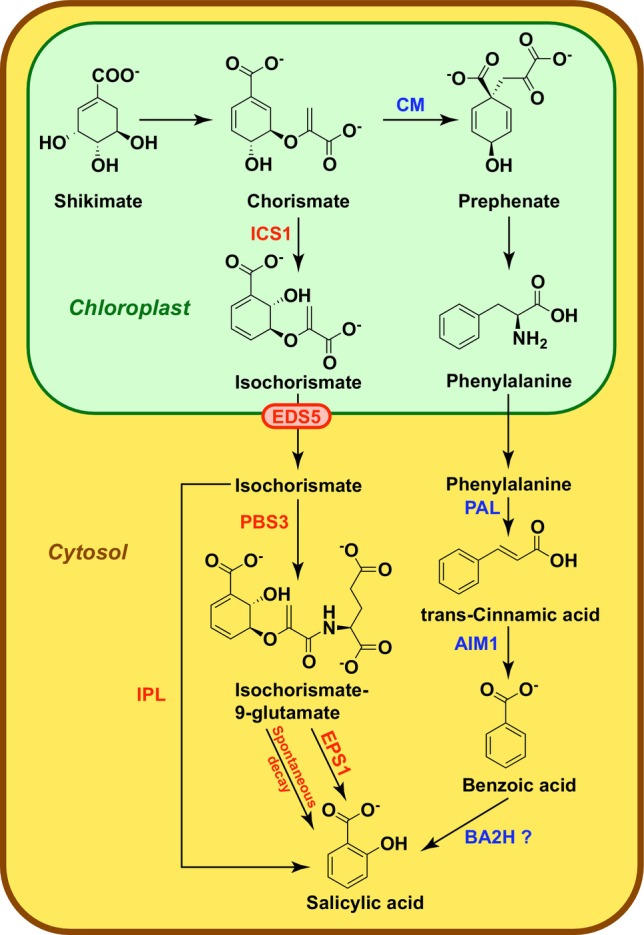
Salicylic acid biosynthesis pathways (adapted from Lefevere *et al*.^13^ and Elsisi *et al*.^49^). ICS1, isochorismate synthase 1; EDS5: enhanced disease susceptibility 5; PBS3: avrPphB Susceptible 3; EPS1: enhanced pseudomonas susceptibility 1; IPL: isochorismate pyruvate lyase; CM: chorismate mutase; PAL: phenylalanine ammonia‐lyase; AIM1: abnormal inflorescence meristem 1; BA2H, benzoic acid 2‐hydroxylase.

SA, or 2‐hydroxybenzoic acid, is a small aromatic compound (C₇H₆O₃) with hydroxyl and carboxyl groups (Fig. 1) that allow hydrogen bonding and solubility in polar solvents.^31^ Its chemical reactivity has been exploited for the synthesis of many derivatives with specific properties.^32^ Despite its widespread use in pharmacology^33^ it is classified as a category 2 CMR compound due to concerns about its reproductive and mutagenic effects.

In plants, SA regulates various biological processes, including morphological development, flowering, and responses to biotic and abiotic stress.^34^, ^35^ It interacts with other hormones such as auxin, influencing root morphology,^36^ enhancing seed germination under abiotic stress, and promotes tolerance to salinity and heat. For instance, SA application improved broad bean (*Vicia faba* L.) seed germination under saline conditions^37^ and increased rice (*Oryza sativa*) growth and yield under thermal stress.^38^ It also contributes to photosynthetic efficiency under cadmium stress by modulating RuBisCO (ribulose‐1,5‐biphosphate carboxylase/oxygenase) activity, a key enzyme in photosynthesis.^39^

The key role of SA in plant defense was first reported when SA and aspirin induced resistance of tobacco against TMV.^40^ Later studies confirmed that SA accumulation is essential for resistance to fungi, bacteria, viruses, and even insects, and is closely associated with SAR and *PR* gene activation.^41^, ^42^

SA is synthesized from chorismate, a product of the shikimate pathway, through two main routes: the isochorismate synthase (ICS) pathway and the phenylalanine ammonia‐lyase (PAL) pathway (Fig. 1).^43^ The ICS pathway is predominant in *Arabidopsis thaliana* plants (Thale cress), where two ICSs (ICS1 and ICS2) have been identified. ICS1 is responsible for the production of approximately 90% pathogen‐induced SA.^44^, ^45^ Chorismate is converted to isochorismate in chloroplasts and exported to the cytosol *via* the enhanced disease susceptibility 5 protein (EDS5).^46^, ^47^ Subsequently, isochorismate is conjugated to L‐glutamate by the acyl‐adenylase AvrPphB Susceptible 3 (PBS3), resulting in the formation of isochorismate‐9‐glutamate,^48^ which undergoes spontaneous degradation into SA in almost all plant species. In the *Brassicaceae* family, the enhanced Pseudomonas susceptibility 1 acyltransferase (EPS1) contributes to efficient release of SA.^48^, ^49^

The synthesis of SA and the resistance to pathogens were shown to be strongly reduced in mutants lacking functional ICS1.^50^ However, the presence of residual SA in a double mutant *ics1 ics2* confirms that the ICS pathway is not the only one available for SA biosynthesis in this plant.^44^

In the PAL pathway, chorismate is converted to phenylalanine *via* chorismate mutase (CM) and a series of chemical reactions, then to trans‐cinnamic acid by PAL.^13^ This intermediate is oxidized to benzoic acid *via* abnormal inflorescence meristem 1 enzyme (AIM1), which is then hydroxylated into SA presumably by benzoic acid 2‐hydroxylase (BA2H).^8^, ^13^ Although ICS is the major contributor, residual SA in *ics1 ics2* double mutant confirms that both pathways contribute to total SA production, with relative importance depending on plant species.^44^

## 4 A SIMPLE MOLECULE WITH COMPLEX METABOLISM: SALICYLIC ACID DERIVATIVES *IN PLANTA*

**Figure 2:**
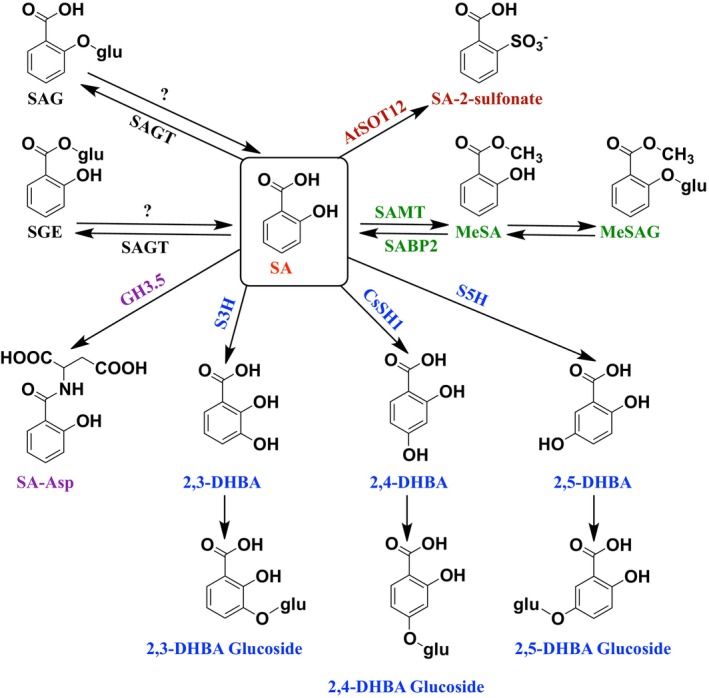
Salicylic acid metabolism in plants (adapted from Vlot *et al*.^8^, Lu *et al*.^51^ and Peng *et al*.^52^). SA, salicylic acid; SAG, SA O‐β‐glucoside; SGE, salicyloyl glucose ester; SAGT, SA glucosyltransferase; SA‐Asp, salicyloyl aspartate; GH3.5, acyl‐amido synthetase Gretchen Hagen 3; 2,3‐DHBA, 2,3‐dihydroxybenzoic acid; S3H, SA 3‐hydroxylase; 2,4‐DHBA, 2,4‐dihydroxybenzoic acid; CsSH1, *C. sinensis* SA 4/5‐hydroxylase; 2,5‐DHBA, 2,5‐dihydroxybenzoic acid; S5H, SA 5‐hydroxylase; MeSA, methyl salicylate; SAMT, SA methyltransferase; SABP2, SA‐binding protein 2; MeSAG, methyl salicylate O‐β‐glucoside; AtSOT12, *A. thaliana* sulfotransferase.

Following biosynthesis, SA undergoes numerous modifications that generate various derivatives and conjugates. The best‐known are glucose or amino acid conjugates, but hydroxylated, sulfonated, or methylated forms are also common (Fig. 2).^8^, ^51^, ^52^

Most SA in plants is converted to SA 2‐O‐β‐D‐glucose (SAG) *via* glycosylation of the phenolic group by salicylic acid glucosyltransferases (SAGTs).^53^, ^54^ In *A. thaliana*, two SAGTs were identified: one mainly forms SAG, while the other produces salicyloyl glucose ester (SGE) through glycosylation of the carboxyl group.^55^ Both conjugates are stored in vacuoles in inactive form and can be hydrolyzed back to SA to activate defense responses.^53^, ^56^

Conjugation with amino acids is less studied. To the best of our knowledge, only salicyloyl‐aspartate (SA‐Asp) has been described in common bean (*Phaseolus vulgaris* L.) and in *A. thaliana*, probably catalyzed by GH3.5 of the GH3 acyl‐amido synthase family.^57^, ^58^, ^59^ Unlike SAG, SA‐Asp does not revert to free SA but functions as a mobile signal for *PR1* gene induction, and resistance against *Pseudomonas syringae*.^58^

Hydroxylated derivatives of SA include 2,3‐dihydroxybenzoic acid (2,3‐DHBA), 2,4‐DHBA, and 2,5‐DHBA (gentisic acid).^51^, ^60^ In *Arabidopsis*, 2,3‐DHBA and 2,5‐DHBA are synthesized by the SA 3‐hydroxylase (S3H) and the SA 5‐hydroxylase (S5H), respectively, often at higher levels than SA itself, mostly as sugar conjugates.^51^, ^61^ These acids and their glycosylated forms accumulate in *Arabidopsis* upon pathogen infection.^51^ In tomato, 2,5‐DHBA is strongly induced by citrus exocortis viroid and TMV, suggesting a signaling role distinct from SA, as it activates a subset of *PR* genes not responsive to SA.^8^, ^51^ A recent study has revealed the existence of 2,4‐DHBA and its glycosylated form as native derivatives of SA in plants.^51^ In tea (*Camellia sinensis*), SA is converted to 2,4‐DHBA by CsSH1 and then glycosylated by UDP‐glucosyltransferase UGT95B17. Infection by the phytopathogenic bacterium *Pseudopestalotiopsis camelliae‐sinensis* enhances 2,4‐DHBA accumulation, which may contribute to basal resistance.^51^

Another well‐studied derivative is methyl salicylate (MeSA), formed by salicylate methyltransferase (SAMT). MeSA accumulates in tobacco following infection by TMV,^62^ and moves to systemic tissues, where it can be reconverted to SA by SABP2 esterase (SA‐binding protein 2), thereby contributing to SAR.^29^, ^63^ However, its role is debated, as SAR can still occur in mutants of *A. thaliana* unable to produce MeSA.^64^ Later studies showed that the requirement of MeSA for SAR induction depends on light conditions during infection by the pathogen.^65^ A glycosylated form, MeSAG, has also been identified as a possible inactive storage form.^66^

Sulfonated SA has also been described. Overexpression of the sulfotransferase *AtSOT12* in *A. thaliana* confirmed its ability to sulfonate the phenolic group SA, leading to increased SA levels and enhanced resistance to *P. syringae*. However, the precise role of sulfonated SA in signaling and SAR remains unclear.^67^

Together, these metabolic conversions highlight the complexity of SA regulation *in planta*, where derivatives can serve as inactive storage forms, transportable signals, or bioactive defense molecules. Importantly, this metabolic diversity is mirrored by the multiplicity of SA targets and receptors. As reviewed by Klessig *et al*.,^68^ SA does not act through a single pathway but binds to a wide array of proteins—including NPR1, NPR3/4, catalases, thioredoxins, and methylesterases—that collectively shape the outcome of defense signaling. This reinforces the notion that the biological functions of SA derivatives must be interpreted within a multi‐target framework, where chemical diversity translates into signaling complexity.

Synthetic SA analogs mimicking these functions are presented in the next section.

## 5 SALICYLIC ACID ANALOGS AS INDUCERS OF PLANT DEFENSE MECHANISMS

The crucial role of SA in plant defense has been confirmed repeatedly through exogenous applications, which can enhance resistance against a variety of pathogens. For example, SA treatment of barrel medic (*Medicago truncatula*) induced the expression of *PR1*, *PR5*, and *PR10* genes and increased resistance against the bacterium *Ralstonia solanacearum*.^69^ In tomato, exogenous SA improved resistance to tomato yellow leaf curl virus (TYLCV) through induction of PR proteins and detoxifying enzymes such as superoxide dismutase (SOD), peroxidase (POD), and ascorbate peroxidase (APX).^70^ Nevertheless, the rapid metabolism, compartmentalization, and potential phytotoxicity of SA limit its long‐term efficacy.^13^, ^71^

In agricultural practice, SA analogs such as acibenzolar‐S‐methyl (BION®/Actigard®) and probenazole (Oryzemate®) have already been deployed, typically providing protection for about 1 to 2 weeks in crops like rice, vegetables, and fruit trees. While these activators reduce disease symptoms and can be integrated into management programs, their efficacy is often partial and requires repeated applications, sometimes with fitness or residue concerns.^14^, ^72^, ^73^, ^74^, ^75^, ^76^

To overcome these drawbacks, synthetic defense inducers have been developed to mimic the activity of SA while improving stability, bioavailability, and persistence (Table 1). These analogs have also provided useful tools to probe SA signaling mechanisms.

**Table 1:** Synthetic salicylic acid analogs and their effect on the innate plant defense system for different pathosystems

Chemical structure and name	Plants/Pathogens	Stimulated defenses	References
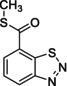 **Acibenzolar‐S‐methyl Benzothiadiazole (BTH)**	Tobacco / Tobacco mosaic virus, *Cercospora nicotianae*, *Erwinia carotovora* Tomato / Cucumber mosaic virus Apple tree */ Erwinia amylovora*	Induction of the *PR* genes and SAR.	Bektas and Eulgem, (2015)^14^ Anfoka, (2000)^80^ Dugé de Bernonville *et al*., (2014)^72^
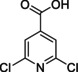 **2,6‐Dichloroisonicotinic acid (INA)**	Common bean / *Pseudomonas syringae pv. Phaseolicola*	Potentialization of the induction of genes involved in defense. Remodeling of the cell wall to increase plant resistance to enzymatic hydrolysis.	Martínez‐Aguilar *et al*., (2016)^81^ De la Rubia *et al*., (2021)^82^
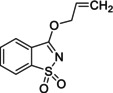 **Probenazole (PBZ)**	Rice / *Magnaporthe grisea*, *Xanthomonas oryzae pv. Oryzae* Arabidopsis / *Pseudomonas syringae pv. tomato DC 3000*, *Peronospora parasitica Emco5*	Induction of the expression of several defense genes, including the *PR* gene. Induction of an accumulation of SA and SAR.	Zhu *et al*., (2024)^78^ Wu *et al*., (2021)^86^ Yoshioka *et al*., (2001)^84^ Nakashita *et al*., (2002)^85^
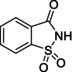 **Saccharine**	Rice / *Magnaporthe grisea*, *Xanthomonas oryzae pv. Oryzae* Wheat / *Blumeria graminis f. sp. Tritici* Kiwi de Chine / *P. syringae pv. actinidiae*	Induction of the SAR in rice. Induction of the expression of several defense‐related genes, including *PR*, *NPR1*, *PAL* and *WRKY*. Accumulation of SA.	Walters *et al*., (2013)^75^ Phuong *et al*., (2020)^88^ Mejri *et al*., (2021)^89^ Reglinski *et al*., (2024)^74^
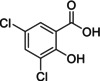 **3,5‐dichlorosalicylic acid (3,5‐DiClSA)**	Tobacco / Tobacco mosaic virus Apple tree / *Venturia inaequalis*	Induction of *PR1* gene expression. Metabolic reprogramming associated with defense and resistance.	Conrath *et al*., (1995)^90^ Lateur, (2002)^91^ Hamany Djande *et al*., (2023)^92^

### 5.1 Acibenzolar‐S‐methyl

Acibenzolar‐S‐methyl (CAS name *S*‐Methyl 1,2,3‐benzothiadiazole‐7‐carbothioate; CAS number 135158–54‐2), commonly referred to as benzothidiazole (BTH), is a functional analog of SA identified through SAR‐inducer.^14^ The molecular structure and chemical class of benzothiadiazole plant activators were described by Kunz *et al*., highlighting the key structural elements responsible for their biological activity.^77^ BTH activates the SA/NPR1 pathway and induces *PR* gene expression, thereby conferring resistance against viruses, fungi, and bacteria such as TMV, *Cercospora nicotianae*, and *Erwinia carotovora*.^78^

BTH is considered a prodrug that undergoes hydrolysis *in planta*, converting its thioester function into a carboxyl group and thereby yielding the biologically active metabolite, acibenzolar acid (CAS name 1,2,3‐Benzothiadiazole‐7‐carboxylic acid; CAS number 35272–27‐6).^79^ Acibenzolar acid is required for SAR induction, as demonstrated in tobacco SABP2‐deficient plants, where BTH failed to activate defenses while acibenzolar acid remained effective.^79^

BTH itself has no direct antimicrobial activity, but primes plant signal transduction as observed in tomato resistance to a strain of cucumber mosaic virus^80^ and in apple resistance to fire blight *Erwinia amylovora*.^72^ Syngenta commercialized BTH under the brand names BION®50WG in Europe and Actigard® in the United States, which have been widely used for protection against rice blast and powdery mildews since the early 1990s.^14^ However, approval for BTH was revoked by the European Commission, and its use was banned in Europe starting from July 2025.

### 5.2 2,6‐Dichloroisonicotinic acid

2,6‐dichloroisonicotinic acid (INA; CAS name 2,6‐dichloropyridine‐4‐carboxylic acid; CAS number 5398‐44‐7) was another product of SAR‐inducer screening. INA induces resistance in cucumber (*Cucumis sativus*) against the fungal pathogen *Colletotrichum lagenarium* ^14^ and enhances the induction of *WRKY29* and *WRKY53* gene expression in common bean in response to the bacterium *P. syringae* pv. *Phaseolicola*.^81^ In the same pathosystem, pre‐treatment with INA was also linked to improved cell wall remodeling, thereby strengthening plant resistance to cell wall‐degrading enzyme activity.^82^

INA induces SAR in tobacco and *Arabidopsis* in a manner similar to SA, but increases SA levels and is effective even in NahG plants, suggesting it functions downstream of SA and partly independent of NPR1.^78^ Its phytotoxicity, however, restricts its use to laboratory studies.^83^

### 5.3 Probenazole

Probenazole (PBZ; CAS name 1,2‐benzisothiazole, 3‐(2‐propen‐1‐yloxy)‐1,1‐dioxide; CAS number 27605–76‐1) is a benzisothiazole derivative first identified in Japan for protection against rice blast (*Magnaporthe grisea*).^76^ PBZ was later commercialized as Oryzemate® and has also been effective against bacterial leaf blight (*Xanthomonas oryzae* pv. *Oryzae*).^78^

PBZ and its active metabolite, 1,2‐benzisothiazole‐1,1‐dioxide (BIT; CAS number 5669‐05‐6), induce the expression of *PR* genes and SAR, but requires both SA and NPR1 in *Arabidopsis* and tobacco.^84^, ^85^ Mutant studies confirmed that PBZ and BIT act upstream of SA/NPR1 signaling. Recent metabolomic analyses revealed that PBZ reshaped rice metabolism, increasing SA, shikimate, and several defense‐related metabolites while lowering phenylalanine and proline levels.^86^ This reprogramming is consistent with earlier observations of PBZ‐induced resistance to *M. grisea* in rice mediated by SA accumulation.^87^ PBZ remains an important crop protection tool for rice, and its broader potential for other crops continues to be investigated.

### 5.4 Saccharine

Saccharine (CAS name 1,2‐benzisothiazol‐3(*2H*)‐one 1,1‐dioxide; CAS number 81–07‐2) is both an artificial sweetener and a PBZ byproduct. It has been shown to induce SAR in rice against *M. grisea* and *X. oryzae*, and in faba bean, soybean and barley against various pathogens.^75^, ^88^, ^89^

Saccharine triggers *PR*, *NPR1*, *PAL*, and *WRKY* gene expression, enhancing defense without direct antifungal activity. Field and greenhouse studies in kiwi (*Actinidia chinensis*) confirmed that saccharine enhances resistance against *P. syringae* pv. *actinidiae* by stimulating SA/SAG accumulation and *PR1* and *PR2* gene expression.^74^ However, saccharine residues exceeding maximum residue levels (MRLs) have been detected in fruit, limiting its practical use in disease management.

### 5.5 Halogenated derivatives of SA

Halogenated salicylates, including 4‐chloro‐, 5‐chloro‐, and 3,5‐dichlorosalicylic acid (3,5‐DiClSA), act as synthetic elicitors. They induce the expression of *PR1* genes and enhance tobacco resistance against TMV.^90^ Additionally, the 3,5‐DiClSA reduced apple scab (*Venturia inaequalis*) by approximately 56% when applied preventively.^91^

Recent metabolomic profiling of barley treated with 3,5‐DiClSA showed significant shifts in primary and secondary metabolism, notably activation of the phenylpropanoid pathway, a hallmark of induced resistance.^92^ While not yet used in crop protection, such findings illustrate how halogenated derivatives reprogram metabolism to strengthen defense responses.

Beyond the compounds described above, a wide range of other functional analogs and activators of the SA pathway have been reported. These have been classified either according to their chemical structure^73^, ^93^ or based on their mode of action within the plant.^93^ For instance, molecules such as Validamycin A (also known as Validoxylamine A), imprimatins, and other ‘upstream activators’ that modulate defense responses before or during SA biosynthesis have been identified in recent studies.^93^ Given the indirect mechanisms of action of many of these compounds, appropriate screening strategies are required to reveal their immunostimulatory properties. Such approaches not only improve the identification of novel candidates but also broaden the exploration of underexploited chemical spaces for the development of innovative plant defense stimulators.^73^, ^93^

## 6 FROM SALICYLIC ACID TO SMART DELIVERY: EMERGING STRATEGIES FOR NATURAL DEFENSE STIMULATION

A number of strategies based on SA have been developed with the aim of maintaining optimal long‐term activity, including the development of nanoparticles coupled to SA, conjugates of SA with other molecules and vectorization systems for targeted transport within plant tissues. The primary constraints to the widespread agricultural use of SA remain in its phytotoxicity and its rapid compartmentalization. These approaches therefore aim to enhance exogenous SA efficiency by ensuring controlled, gradual release and targeted delivery throughout plant tissues, extending defense responses and improving sustainability. Recent research also aims to mimic the natural dynamics of SA metabolism and transport *in planta*, ensuring that the hormone remains bioavailable without reaching toxic levels.^71^, ^94^

Controlled‐release formulations of SA have been shown to sustain the expression of defense marker genes such as *PR1* and *NPR1* for several days longer than direct SA application, thus prolonging resistance.^94^, ^95^ However, maintaining continuous SA signaling may also create trade‐offs with growth and other hormonal pathways, highlighting the need for balanced regulation.^96^, ^97^

**Figure 3:**
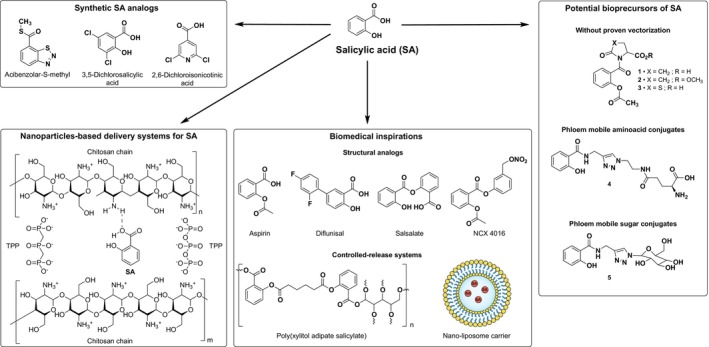
Examples of salicylic acid (SA) analogs and derivatives designed to enhance plant defense responses, together with biomedical concepts that may inspire future developments. Synthetic SA analogs: representative molecules such as acibenzolar‐S‐methyl, 3,5‐dichlorosalicylic acid, and 2,6‐dichloroisonicotinic acid; Nanoparticle‐based delivery systems: hypothetical representation of an SA–chitosan nanocomposite;^94^ Potential bioprecursors of SA: SA conjugates with pyroglutamic acid (1–3)^107^ and vectorized forms with glutamic acid (4) or glucose (5);^114^ Biomedical inspirations: examples from the medical field — including structural analogs (aspirin, diflunisal, salsalate, NO‐releasing salicylates) and controlled‐release platforms (polymeric or lipid‐based carriers) — proposed as conceptual models for next‐generation SA‐based elicitors in plants.^123^, ^125^, ^126^

The following sections summarize the main innovative strategies currently explored for SA delivery and activation in plants (Fig. 3).

### 6.1 SA nanocomposites

In order to facilitate the coupling of SA to nanoparticles, a variety of both organic and inorganic compounds are employed as supports. Consequently, chitosan, the second most abundant polysaccharide in nature after cellulose, is the most frequently implemented compound as a matrix.^71^ Chitosan, a deacetylated derivative of chitin derived from crustacean shells, possesses amino and hydroxyl groups that allow interaction with acidic molecules such as SA.^98^ Its physicochemical properties depend on the degree of deacetylation (DD) and molecular weight (MW), which influence nanoparticle formation, particle size, surface charge and release kinetics. For example, studies on chitosan‐sodium tripolyphosphate (TPP) nanoparticles showed that higher DD chitosan (85–93%) yields smaller, more uniform particles,^99^ and in alginate/chitosan‐coated systems, low MW and high DD significantly improved encapsulation efficiency and release/bioaccessibility of phenolic compounds (quercetin).^100^

Recent studies have reported the development of a nanocomposite based on chitosan and SA, using ionic gelation in acetic acid solution with TPP as a cross‐linker.^95^, ^101^, ^102^ The acidic environment protonates chitosan amino groups, and TPP bridges polymer chains, forming hydrogen bonds with SA and effectively encapsulating it.^71^ The gradual hydrolysis of glycosidic bonds in neutral or slightly acidic environments results in progressive SA release from the chitosan matrix, providing sustained bioavailability.^95^, ^101^ This sustained delivery prolongs defense gene activation, improving the duration of protection without growth inhibition.^95^

Kumaraswamy *et al*. have demonstrated that foliar application of this SA‐chitosan nanocomposite controlled post‐flowering stalk rot in maize (*Fusarium verticillioides*), while maintaining plant growth.^95^ Likewise, Martin‐Saldaña *et al*. showed that in lettuce, this formulation induced *NPR1* and *PR2* expression, consistent with activation of the SA pathway.^101^ Similar chitosan‐based nanocarriers have been proposed for many agricultural purposes, highlighting their potential for integrated crop protection systems.^103^

Additionally, silica nanoparticles have been used in the design of SA nanocomposites. Silica nanoparticles modified with thiol‐carboxymethyl‐β‐cyclodextrin form disulfide bridges with cystamine that entrap SA.^71^ These linkages respond to plant redox status: glutathione reduces disulfides to release SA, whereas H₂O₂ reforms them, controlling release rates.

In an alternative strategy, mesoporous silica nanoparticles functionalized with decanethiol were used to encapsulate SA within their pores, with the release shown to be dependent on the plant's redox potential.^94^ *Arabidopsis* plants treated with this nanocomposite showed sustained expression of the *PR1* gene for up to 7 days, whereas plants treated with free SA showed a rapid initial peak in *PR1* expression, followed by a decline after 3 days. Thus, this nanocomposite facilitates a slow and stable release of SA, providing sustained protection for the plant against biotic stresses.^94^ In pineapple (*Ananas comosus* L.), similar formulations improved resistance to the fungus *Phytophthora cinnamomi*, as evidenced by reduced lesion development, and enhanced root growth in infected plants when compared to treatment with free SA.^104^

Despite promising results, the practical use of nanocomposites raises concerns regarding their persistence, ecotoxicity and human safety, the environment, and biodiversity. Risk assessment frameworks for nanomaterials in agriculture are still evolving, with regulatory gaps in nanoparticle size, biodegradability, and accumulation potential.^105^, ^106^

### 6.2 SA conjugates with pyroglutamic acid

Inspired by prodrug design in pharmacology, the hypothesis that prodrugs of SA with enhanced elicitation properties and higher bioavailability could be obtained by linking SA with gamma‐aminobutyric acid (GABA) or its analogs led to the development of SA conjugates with pyroglutamic acid (PGA). GABA is a non‐proteinogenic amino acid shown to induce resistance in several crops against *Penicillium expansum* on pear and *Alternaria alternata* or *Botrytis cinerea* on tomato.^107^ PGA, a GABA analog, has not previously been tested in plant defense, but is found in proteins associated with stress responses.^107^

Consequently, five SA‐PGA conjugates were synthesized and tested in wheat against *Zymoseptoria tritici*. Four compounds reduced the incidence of disease symptoms by up to 49% and outperformed SA itself.^107^ *In vitro*, one conjugate showed slight direct antifungal activity, but most induced resistance indirectly, likely *via* endogenous signaling.^107^

However, while the authors have confirmed the hypothesis that these prodrugs can increase the protective activity of the SA, several aspects remain unclear, particularly regarding the defensive pathways involved and the ecotoxicological impacts of their practical application.

### 6.3 Vectorization of SA in plants

The mechanisms of cellular and long‐distance transport of SA and its derivatives in plant tissues remain poorly understood. It was initially assumed that SA uptake by plant cells occurred exclusively *via* an ion‐trapping mechanism due to the presence of its carboxylic acid function.^108^ However, two transporters involved in SA absorption have been identified in humans.^109^ Consequently, following investigations into the transport of SA and its derivatives within the plant, Rocher *et al*. demonstrated that the transport of SA did not solely occur *via* the ion‐trapping mechanism, but also involved an active pH‐dependent transport system.^110^, ^111^ More recently, it has been emphasized that the systemic mobility of SA involves a combination of diffusion, metabolic conversion, and phloem‐mediated redistribution within the plant.^112^

Based on previous work on phloem‐mediated transport of natural and xenobiotic compounds and on emerging strategies for the targeted delivery of active molecules in plants, a specific approach was developed for the vectorization of SA. This strategy aims to improve the systemic translocation and bioavailability of SA by conjugating it with nutrient molecules, typically amino acids or sugars, which can be recognized and transported by endogenous nutrient transport system.

The concept, originally explored with fenpiclonil, a contact fungicide of the phenylpyrrole family,^113^ involves coupling the active compound with a nutrient moiety (α‐amino acid or sugar). The resulting conjugates are then recognized and translocated by the plant's active transport systems, as observed in the castor bean (*Ricinus communis*) model.^114^ This approach enables the targeted movement of active compounds to plant tissues that are otherwise poorly accessible, improving bioavailability and potentially reducing phytotoxicity.^113^, ^115^ Additionally, it is worthy of note that the conjugation of SA with a nutrient will influence its distribution within the plant. A similar strategy had previously been explored with the systemic auxinic herbicide 2,4‐D, whose conjugate with L‐lysine (Lys‐2,4D) accumulated five to 10 times more in the root system of broad bean (*Vicia faba* L.) than the free herbicide, following foliar application.^116^ This finding highlights the potential of nutrient conjugation to reorient systemic transport pathways through the phloem toward specific plant organs.

Guichard *et al*. have further developed six potential vectorized prodrugs (or bioprecursors) of SA and its chlorinated derivatives.^114^ Among them, a conjugate of SA with glutamic acid exhibited phloem mobility in the *Ricinus* model similar to that of free SA, despite its higher steric hindrance. These compounds were subsequently evaluated for their protective efficacy against two maize (*Zea mays* L.) pathogens, *Bipolaris maydis* and *Fusarium graminearum*, using foliar application.^114^ The conjugate showing the best phloem mobility in the *Ricinus* model also provided effective local protection against *B. maydis*. Moreover, it conferred protection against *F. graminearum* inoculated at the stem base, demonstrating long‐distance transport from the leaf application site. In the presence of the fungus *B. maydis*, this bioprecursor induced significant up‐regulation of two defense‐related genes in maize, *ZmNPR1* and *ZmPR1*, confirming its ability to trigger a systemic defense response.^114^

The targeted delivery of SA *via* such bioprecursors therefore represents a promising strategy to stimulate durable plant immunity. However, further characterization of key parameters — such as phloem loading efficiency, metabolic stability, and release kinetics — remains essential before practical implementation. Future work should also examine possible hormonal interactions, since SA signaling is closely interconnected with other defense pathways, notably the jasmonic acid (JA) network — an aspect further discussed in section 6.5.

Building on these plant‐based approaches, inspiration can also be drawn from other scientific fields, particularly medicine, where SA and its analogs have long been studied for controlled and sustained administration.

### 6.4 Biomedical insights for next‐generation SA delivery systems

Although most advances in the controlled delivery of SA have originated in the biomedical field, these developments offer valuable conceptual frameworks for agricultural adaptation. In medicine, SA and its derivatives have long been used for their anti‐inflammatory and antimicrobial properties, but their rapid clearance and local irritation have driven extensive work on sustained‐release formulations. The physicochemical principles underlying these biomedical systems — including biopolymer encapsulation, pH‐ or redox‐responsive matrices, and prodrug‐based release — provide a conceptual foundation for designing future agrochemical formulations capable of maintaining stable and long‐lasting defense activation in plants.

#### 6.4.1 Structural analogs of SA in medicine

In the biomedical field, a wide variety of structural analogs and derivatives of SA have been developed to optimize pharmacokinetic behavior, tissue‐targeting, and controlled‐release profiles. Classic examples include acetylsalicylic acid (aspirin), an acetylated form of SA and one of the most widely used non‐steroidal anti‐inflammatory drugs. Importantly, aspirin has also been studied in plant systems for its capacity to trigger defense responses,^40^, ^117^ underscoring the cross‐kingdom relevance of the salicylate scaffold.

Other analogs such as diflunisal (2′,4′‐difluoro‐4‐hydroxy‐[1,1′‐biphenyl]‐3‐carboxylic acid) exhibit enhanced stability and longer plasma half‐life,^118^ while salsalate (a salicylic acid dimer) produces a relatively flat plasma salicylate profile following administration in rodents.^119^

Furthermore, examples of NO‐releasing aspirin derivatives have been developed in biomedicine: NCX‐4016 combines an aspirin moiety with a nitric oxide donor to exert anti‐inflammatory, anti‐platelet, and gastric‐protective effects in animal and cellular models.^120^ Meanwhile, nitro‐substituted salicylates (e.g., salicylanilides with nitro groups) have been synthesized and tested for antimicrobial and cytotoxic activity *in vitro*, showing that substitution of the salicylate scaffold can modulate biological activity significantly.^121^

These examples illustrate the chemical versatility of the SA scaffold and its potential to inspire next‐generation elicitors in plants, by modulating substituents to control activity, mobility or tissue specificity, a concept still largely unexplored in agronomy.

#### 6.4.2 Controlled‐release systems in biomedical applications

##### 6.4.2.1 Polymeric prodrugs

Polymers incorporating SA monomers within their backbone act as polymeric prodrugs that degrade slowly, releasing SA as the chain hydrolyses. These systems give linear, sustained‐release profiles and were developed originally for long‐term anti‐inflammatory implants or topical devices. As examples of this strategy, Chandorkar *et al*. synthesized cross‐linked SA‐polyesters that released SA over several months *in vitro*,^122^ and Dasgupta *et al*. developed tunable poly(xylitol adipate salicylate) networks that control SA release kinetics.^123^ Such degradable polymer matrices could, by analogy, be adapted in agronomy to deliver SA progressively in the rhizosphere or phyllosphere, limiting phytotoxic peaks.

##### 6.4.2.2 Poly(lactic‐co‐glycolic) acid (PLGA) and other biodegradable microparticles

PLGA micro/nanoparticles and hybrid scaffolds permit controlled diffusion and erosion‐driven release, a concept extensively applied to aspirin or SA derivatives in drug delivery. Chen *et al*. demonstrated that PLGA‐based microspheres and PLGA–CaSiO₃ composite scaffolds released ~95% of encapsulated aspirin within 24 to 36 days *in vitro*, illustrating how formulation parameters can tune release duration.^124^ These well‐characterized systems could be adapted to plant applications, provided that issues of uptake, persistence, and environmental safety are addressed.

##### 6.4.2.3 Lipid‐based carriers

Lipid vehicles are widely employed for poorly water‐soluble molecules and for topical or transdermal delivery. Aspirin and related drugs have been encapsulated in liposomes, solid lipid nanoparticles (SLNs) and nanostructured lipid carriers (NLCs) to minimize burst release, improve chemical stability and modulate tissue absorption.^125^, ^126^ By analogy, such systems might enhance foliar uptake and reduce SA phytotoxicity while providing gradual surface release in crops.

Together, these biomedical systems illustrate a continuum of innovation, from structural analogs (aspirin, diflunisal, NO‐releasing salicylates) to sophisticated polymeric, particulate, or lipid carriers, all sharing the goal of extending activity and controlling site‐specific delivery. Translating these approaches to plant systems will require adapting material biocompatibility, degradation rates, and release triggers (pH, light, enzymes) to the plant microenvironment. Nevertheless, they offer, in addition to innovations developed specifically for the agronomic field, a solid conceptual and technological basis for next‐generation elicitors based on SA.

Overall, the biomedical perspective complements agricultural research by offering mature engineering concepts and material platforms that could be adapted to plant environments. This interdisciplinary convergence highlights the need to integrate such designs with plant hormonal homeostasis and safety considerations, which are discussed in the next section.

### 6.5 Integrating SA homeostasis, hormonal signaling, and biosafety considerations

SA functions within a tightly regulated homeostatic network. SA levels, their compartmentalization and interconversion into inactive or mobile derivatives (e.g., glycosides, MeSA, hydroxylated forms) are controlled by biosynthetic, conjugative and catabolic enzymes, and by transport processes. These mechanisms together determine both the amplitude and the duration of SA‐driven responses.^127^

Since SA signaling is closely interconnected with other hormonal pathways, particularly JA, strategies that increase SA (for example sustained‐release formulations) risk disrupting the balance between defense programs. The classical antagonism between SA‐ and JA‐mediated pathways is well documented and can alter defense mechanisms in favor of resistance to biotrophs, to the detriment of necrotroph/herbivore responses; this could have obvious practical implications when a formulation is used in multi‐threat field situations.^97^, ^128^

Persistent activation of SA signaling can also involve metabolic costs: sustained transcriptional reprogramming, production of PR proteins and specialized metabolites, and reinforcement of cell walls consume carbon, nitrogen and energy, and may reduce resources available for growth and yield. The growth‐immunity trade‐off depends on context, but substantial evidence shows that high SA levels can drive plants into a biased defense state; important exceptions and genetic solutions dissociating defense and growth are described, indicating that management practices or selection can mitigate such costs.^129^

Absolute SA concentrations and the sensitivity of downstream signaling differ considerably depending on the species, stage of development, and tissue. Consequently, the same input dose or release profile will not produce the same results from one crop to another. This interspecific variation argues in favor of crop‐specific optimization (dose, formulation, timing) rather than a one‐size‐fits‐all approach.^127^

Beyond these physiological considerations, practical implementation also depends on compliance with emerging environmental and safety frameworks. From a regulatory and practical standpoint, nano and controlled‐release platforms present both opportunities and obligations. Controlled‐release SA systems can reduce phytotoxic peaks and prolong effective exposure, but regulatory authorities require detailed characterization of the nanomaterial (particle size distribution, surface chemistry, dissolution or aggregation behavior), as well as its environmental fate, ecotoxicity, and potential residues in edible matrices. The EFSA guidance on nanomaterials^130^ and pragmatic regulatory frameworks for nanopesticides^131^ provide useful reference points for data requirements; early dialog with regulators is therefore recommended when developing nano‐derived SA formulations.

## 7 CONCLUSION AND FUTURE PERSPECTIVES

SA plays a central role in the establishment of plant defense mechanisms, making it an excellent natural elicitor and a promising alternative to synthetic pesticides. While exogenous applications can effectively trigger SAR, their efficacy depends strongly on formulation and delivery, since free SA is often phytotoxic and rapidly metabolized or compartmentalized, limiting its long‐term effect.

Recent advances described in this review — including nanocomposites, conjugates, and vectorized bioprecursors — illustrate the potential of new‐generation formulations to ensure gradual release and spatially controlled activation of SA. These approaches may sustain the expression of key defense regulators such as *PR1* and *NPR1*, while reducing the metabolic burden associated with repeated stress induction. However, maintaining prolonged hormonal activation raises questions about possible trade‐offs between growth and defense, and about hormonal crosstalk, particularly between SA and JA pathways, which coordinate responses to biotrophic and necrotrophic pathogens, respectively. Future work should address these aspects experimentally across different crop species.

Nevertheless, beyond biological optimization, regulatory and practical considerations will determine the adoption of SA‐based elicitors in sustainable agriculture. Field‐scale studies under variable environmental conditions remain essential to assess efficacy, persistence, and phytotoxic thresholds. In addition, a comprehensive risk assessment will be essential to evaluate and control potential ecotoxicological effects, including impacts on biodiversity, soil and microbiome health, and compatibility with integrated pest management frameworks, to ensure the safe large‐scale use of SA‐based elicitors.

Interdisciplinary progress combining plant physiology, materials science, and green chemistry offers a promising route toward safe and efficient SA‐based defense stimulators. Together with advances in chemical engineering, high‐throughput screening, and regulatory innovation, these developments could accelerate the transition to next‐generation biostimulants capable of enhancing crop resilience while reducing dependence on conventional pesticides.

## Data Availability

This manuscript is a review, so there are no experimental data.
